# Oxidative Stress Promotes Liver Cancer Metastasis via RNF25‐Mediated E‐Cadherin Protein Degradation

**DOI:** 10.1002/advs.202306929

**Published:** 2024-01-29

**Authors:** Zhao Huang, Li Zhou, Jiufei Duan, Siyuan Qin, Jingwen Jiang, Haining Chen, Kui Wang, Rui Liu, Minlan Yuan, Xiangdong Tang, Edouard C. Nice, Yuquan Wei, Wei Zhang, Canhua Huang

**Affiliations:** ^1^ Department of Biotherapy Cancer Center and State Key Laboratory of Biotherapy West China Hospital Sichuan University Chengdu 610041 China; ^2^ Key Laboratory of Molecular Biology for Infectious Diseases (Ministry of Education) Institute for Viral Hepatitis Department of Infectious Diseases The Second Affiliated Hospital Chongqing Medical University Chongqing 400016 China; ^3^ West China School of Public Health and West China Fourth Hospital Sichuan University Chengdu 610041 China; ^4^ Colorectal Cancer Center Department of General Surgery West China Hospital Sichuan University Chengdu 610041 China; ^5^ West China School of Basic Medical Sciences & Forensic Medicine State Key Laboratory of Biotherapy West China Hospital Sichuan University Chengdu 610041 China; ^6^ State Key Laboratory of Oral Diseases National Clinical Research Center for Oral Diseases Research Unit of Oral Carcinogenesis and Management Chinese Academy of Medical Sciences West China Hospital of Stomatology Sichuan University Chengdu 610041 China; ^7^ Mental Health Center and Psychiatric Laboratory The State Key Laboratory of Biotherapy West China Biomedical Big Data Center West China Hospital of Sichuan University Chengdu 610041 China; ^8^ Sleep Medicine Center Department of Respiratory and Critical Care Medicine Mental Health Center Translational Neuroscience Center State Key Laboratory of Biotherapy West China Hospital Sichuan University Chengdu 610041 China; ^9^ Department of Biochemistry and Molecular Biology Monash University Clayton VIC 3167 Australia; ^10^ Frontiers Medical Center Tianfu Jincheng Laboratory Chengdu 610212 China; ^11^ Medical Big Data Center Sichuan University Chengdu 610041 China

**Keywords:** cancer metastasis, oxidative stress, post‐translational modifications, redox signaling

## Abstract

Loss of E‐cadherin (ECAD) is required in tumor metastasis. Protein degradation of ECAD in response to oxidative stress is found in metastasis of hepatocellular carcinoma (HCC) and is independent of transcriptional repression as usually known. Mechanistically, protein kinase A (PKA) senses oxidative stress by redox modification in its β catalytic subunit (PRKACB) at Cys200 and Cys344. The activation of PKA kinase activity subsequently induces RNF25 phosphorylation at Ser450 to initiate RNF25‐catalyzed degradation of ECAD. Functionally, RNF25 repression induces ECAD protein expression and inhibits HCC metastasis in vitro and in vivo. Altogether, these results indicate that RNF25 is a critical regulator of ECAD protein turnover, and PKA is a necessary redox sensor to enable this process. This study provides some mechanistic insight into how oxidative stress‐induced ECAD degradation promotes tumor metastasis of HCC.

## Graphical abstract

Oxidative stress‐induced E‐cadherin protein degradation is crucial in hepatocellular carcinoma (HCC) metastasis. Phosphorylation of RNF25 is required in the ROS‐induced E‐cadherin degradation. Redox modification enhances PKA kinase activity to phosphorylate RNF25. Disruption of PKA‐RNF25‐ECAD axis is sufficient to inhibit HCC metastasis.

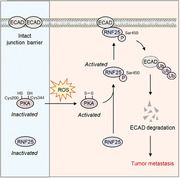

## 1 Introduction

Metastasis is frequently observed in advanced cancer cases and is a major challenge for cancer therapy. Compelling studies have reported that reactive oxygen species (ROS) impede metastasis by inducing death in detached cancer cells. Oxidative stress therefore represents a barrier to the metastatic spread of cancer.^[^ ^1^ ^]^ However, conflicting studies have reported that ROS can also promote metastasis.^[^ ^2^, ^3^, ^4^ ^]^ For instance, ROS has been demonstrated to initiate the epithelial‐to‐mesenchymal transition (EMT) program, a critical process in cancer cell metastasis.^[^ ^2^, ^3^ ^]^ Additionally, ROS have been shown to promote the metastasis of melanoma cells in the aged microenvironment.^[^ ^4^ ^]^ However, the mechanism underlying ROS‐induced cancer metastasis is not yet fully understood.

Adherens junctions are a vast superfamily of calcium‐dependent cell adhesion proteins that are crucial for tissue morphogenesis and development.^[^ ^5^ ^]^ The interactions between adherens junctions and other cellular junctions maintain the structural integrity of solid tissues and control the reconfiguration and recycling of tissue structures.^[^ ^6^ ^]^ Additionally, adherens junctions also control cell segregation and the emergence of different tissue interfaces throughout development.^[^ ^7^ ^]^ In the neoplastic context, adherens junctions, together with other junctional complexes, comprise a significant barrier for preventing cancer cells from metastasis. Loss of adherens junctions is an early event in metastasis, which has been observed in various cancers. Adherens junctions comprise a protein complex consisting of E‐cadherin (ECAD), α‐catenin, β‐catenin, and p120‐catenin, among which ECAD has been most widely investigated. Downregulation of ECAD has long been demonstrated to promote metastasis.^[^ ^8^ ^]^ Some transcription factors such as ZEB, snail, and twist are well known to repress the expression of ECAD at the transcriptional level. However, it remains unclear how ECAD protein is modified and the role of ECAD protein modifications especially in tumor metastasis. Compared with transcription‐dependent control, the protein degradation process is relatively rapid, thus contributing to a quick adaptation for organisms in response to stress. Importantly, ROS have been shown to repress the expression of ECAD, and antioxidants are capable of maintaining ECAD levels, suggesting oxidative stress and tumor metastasis are interconnected.^[^ ^9^ ^]^

Here, we found that ECAD protein is degraded in response to oxidative stress in hepatocellular carcinoma (HCC). The RING finger protein 25 (RNF25) was identified as a novel E3 ligase of ECAD protein ubiquitination. Mechanistic studies revealed that protein kinase A (PKA) undergoes redox modification in response to ROS and leads to phosphorylation of RNF25. ROS‐induced RNF25 phosphorylation degrades ECAD protein to promote HCC metastasis. Overexpression of RNF25 predicted a poor outcome in HCC patients, suggesting that RNF25 can be a potential therapeutic target in HCC metastasis.

## 2 Results

### 2.1 Oxidative Stress Causes E‐Cadherin Protein Repression in HCC

**Figure 1:**
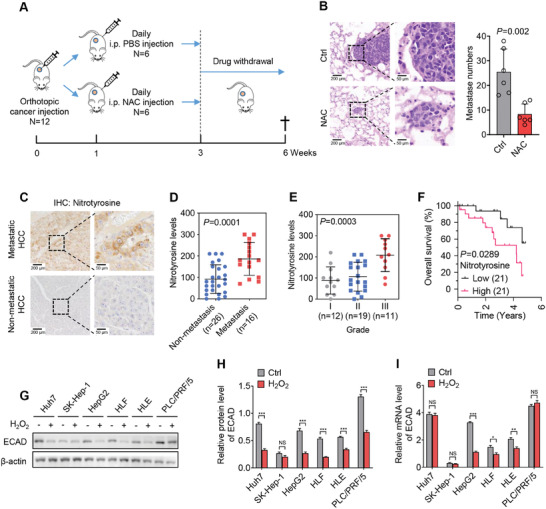
Oxidative stress causes E‐cadherin protein repression in HCC. A) Schematic diagram illustrating the orthotopic mouse model. B) Representative images of H&E staining for the lung metastases in (A). Scale bars, 200 µm (left) or 50 µm (right). C) IHC studies showing the level of nitrotyrosine in human HCC samples. Scale bars, 200 µm (left) or 50 µm (right). D) Data quantification in (C). E) The levels of nitrotyrosine in HCC patients with different grades. F) The overall survival rates of HCC patients with high or low nitrotyrosine levels. G,H) Western blot showing the protein level of ECAD in a panel of HCC cell lines with or without 100 µm H_2_O_2_ treatment for 24 h. I) qPCR assays showing the mRNA level of ECAD in cells treated as (G). Data are mean ± SD from at least three independent repeats. *p*‐value was determined by unpaired *t*‐test (B,D,H,I), one‐way ANOVA (E), and Log‐rank (Mantel‐Cox) test (F). ^*^ *p* < 0.05, ^**^ *p* < 0.01; ^***^ *p* < 0.001. NS, no statistical significance.

To confirm how oxidative stress affects tumor metastasis in vivo, an orthotopic mouse model was established and then treated with the antioxidant N‐acetyl cysteine (NAC). As shown in **Figure** **1**A,B, NAC diminished the development of lung metastases, suggesting that oxidative stress promotes HCC metastasis. In addition, immunohistochemistry (IHC) staining demonstrated a high level of nitrotyrosine, an indicator of oxidative stress, in metastatic human HCC samples (Figure 1C,D). High nitrotyrosine levels were found to correlate with advanced stage and poor survival in HCC patients (Figure 1E,F).

Several independent groups have reported the critical role of ECAD in tumor metastasis.^[^ ^8^, ^10^, ^11^ ^]^ We therefore detected the protein expression of ECAD in H_2_O_2_‐treated HCC cells. As shown, the protein level of ECAD was repressed in H_2_O_2_‐treated HCC cells (Figure 1G,H; Figure S1A, Supporting Information). However, the mRNA level of ECAD was unchanged in Huh7 and PLC/PRF/5 cells after H_2_O_2_ treatment (Figure 1I). Moreover, H_2_O_2_‐mediated ECAD protein repression can be abrogated by the proteasome inhibitor MG132, indicating that H_2_O_2_ inhibits ECAD level via downregulating its protein stability in HCC cells (Figure S1B, Supporting Information). These observations suggested that oxidative stress‐induced ECAD repression is regulated at the protein level in HCC, which is at least partially independent of transcriptional repression. We also detected the nitrotyrosine and ECAD levels in the primary tumors and metastatic tumors in the above mouse model. Surprisingly, no significant difference was observed between primary tumors and metastatic tumors (Figure S1C, Supporting Information). This may be due to the fact that metastatic tumor cells re‐express ECAD and re‐establish the antioxidant system after distant colonization. In addition, the expression of other EMT markers including N‐cadherin (NCAD), ZO‐1, and vimentin was not obviously changed upon H_2_O_2_ treatment, indicating an exclusive role of ECAD in ROS‐driven HCC metastasis (Figure S1D, Supporting Information).

Oxidative stress is tightly linked to pro‐inflammatory cytokines, such as TGFβ, IL‐6, and EGF.^[^ ^12^, ^13^ ^]^ For example, TGFβ can directly induce ROS production in mitochondria.^[^ ^14^ ^]^ To ascertain the repressive effect of ROS on ECAD expression, we treated HCC cells with recombinant TGFβ1, IL‐6, and EGF proteins. As shown, these cytokines induce an EMT morphological change with an increased level of ROS in HCC cells (Figure S1E,F, Supporting Information). Similar to H_2_O_2_, TGFβ1‐induced ROS also repressed the protein level of ECAD (Figure S1G,H, Supporting Information). Moreover, the mRNA level of ECAD was slightly repressed in PLC cells, and unchanged in Huh7 cells (Figure S1I,J, Supporting Information). Together, these data indicate that oxidative stress frequently downregulates ECAD at the protein level in HCC, which may contribute to HCC metastasis.

### 2.2 RNF25 is an E3 Ligase of E‐Cadherin Protein

**Figure 2:**
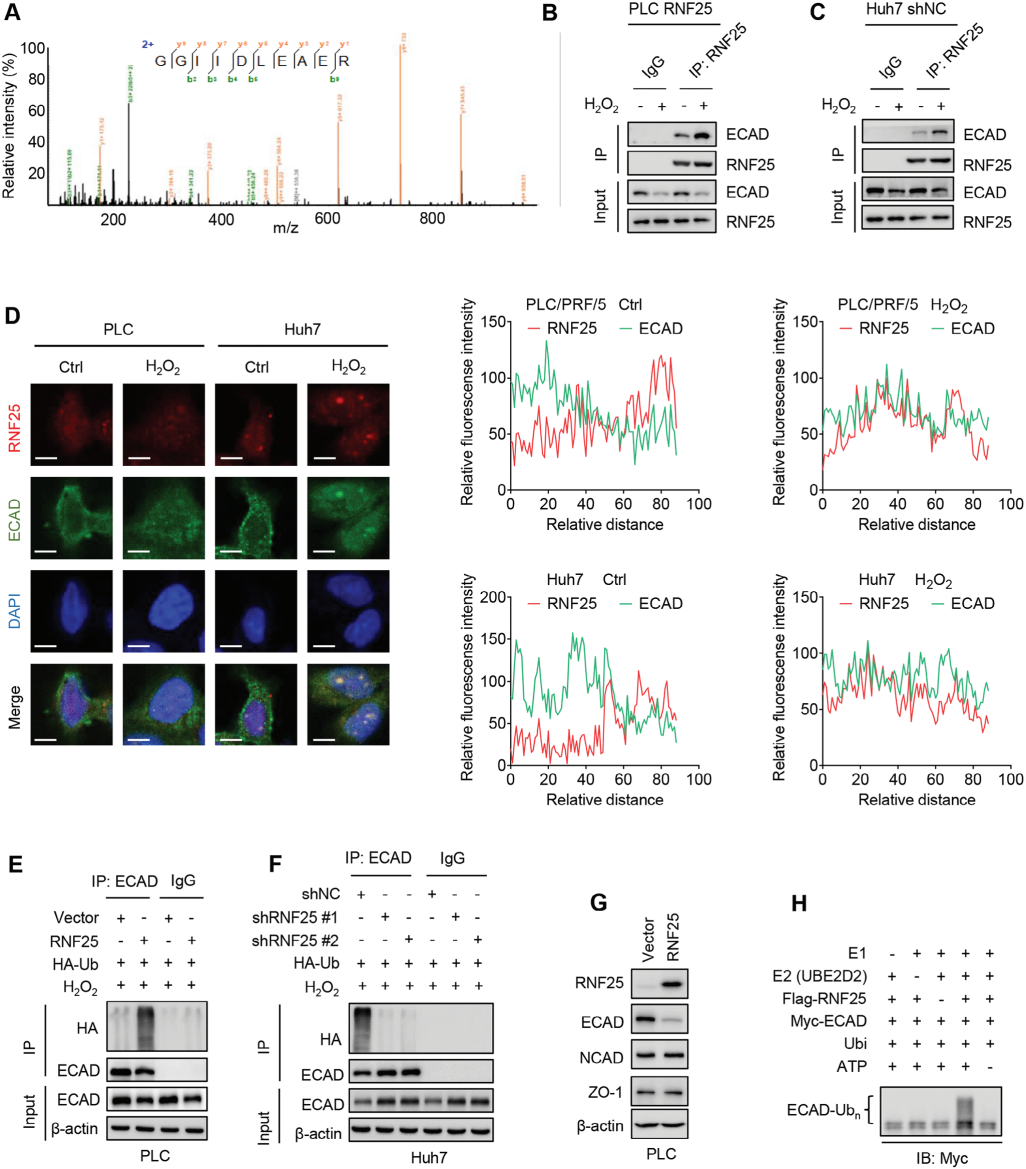
RNF25 is an E3 ligase of E‐cadherin protein. A) MS spectra of RNF25 obtained from ECAD‐interacted proteins by co‐IP. B,C) Co‐IP assays showing the interaction between ECAD and RNF25 using RNF25‐overexpressed PLC stable cells and Huh7 shNC stable cells treated with or without 100 µm H_2_O_2_ for 1 h. D) Immunofluorescence assay showing the subcellular localization of RNF25 (red) and ECAD (green) in HCC cells treated with or without 100 µm H_2_O_2_ for 24 h. Scale bar, 10 µm. E,F) Co‐IP assays showing the ubiquitination of ECAD in indicated stable cells treated with 100 µmH_2_O_2_ for 24 h. G) Western blot showing the expression of indicated proteins in RNF25‐overexpressed cells. H) Ubiquitination of immunoprecipitated ECAD by purified RNF25 in vitro.

Given that the transcription of ECAD remains unchanged, and the protein stability of ECAD is altered, we speculated that oxidative stress represses ECAD by post‐translational modifications (PTMs). To verify this, we analyzed several common PTMs, including phosphorylation, acetylation, and ubiquitination on ECAD under oxidative stress. As shown in Figure S2A (Supporting Information), phosphorylation of ECAD was observed in PLC and Huh7 cells, but this modification was not obviously changed upon H_2_O_2_ treatment. No significant acetylation was detected on ECAD in both cell lines (Figure S2A, Supporting Information). By contrast, the ubiquitination of ECAD was enhanced under oxidative stress (Figure S2A, Supporting Information). We therefore focused on the ubiquitination of ECAD. Then, binding partners of ECAD were screened by co‐IP followed by mass spectrometry (MS). Among the top 10 interactors (Table S1, Supporting Information), the RING finger protein 25 (RNF25) is the only one annotated with an E3 ligase function (**Figure** **2**A). To determine the potential E3 activity of RNF25 to ECAD, we established stable PLC cells with Flag‐tagged RNF25 overexpression and stable Huh7 cells with RNF25 silencing. Both stable cell lines were used to identify the interaction between ECAD and RNF25. As shown in Figure 2B,C, RNF25 was found to interact with ECAD in both HCC cell lines. These interactions were further augmented after H_2_O_2_ stimulation. Additionally, in the absence of oxidative stress, RNF25 and ECAD localize at the cytoplasm and cell membrane, respectively. By contrast, these two proteins are co‐localized at the cytoplasm upon the H_2_O_2_ treatment, indicating that ROS promotes the colocalization and interaction of RNF25 and ECAD (Figure 2D). Furthermore, overexpression of RNF25 activated the ubiquitination of ECAD protein, whereas knockdown of RNF25 exhibited the opposite effect (Figure 2E,F). Consistently, RNF25 downregulates ECAD at the protein but not at the mRNA level (Figure 2G; Figure S2B–D, Supporting Information). Moreover, an in vitro ubiquitination assay also demonstrated that ECAD is ubiquitinated by RNF25 (Figure 2H). In addition, RNF25 was found to be highly expressed in PLC and Huh7 cells but hardly expressed in several other HCC cells including SK‐Hep‐1, HLE, HLF, and HepG2 (Figure S2E, Supporting Information). Together, these results suggest that RNF25 is a novel E3 ligase of ECAD.

### 2.3 Phosphorylated RNF25 at Ser450 Initiates E‐Cadherin Protein Degradation

**Figure 3:**
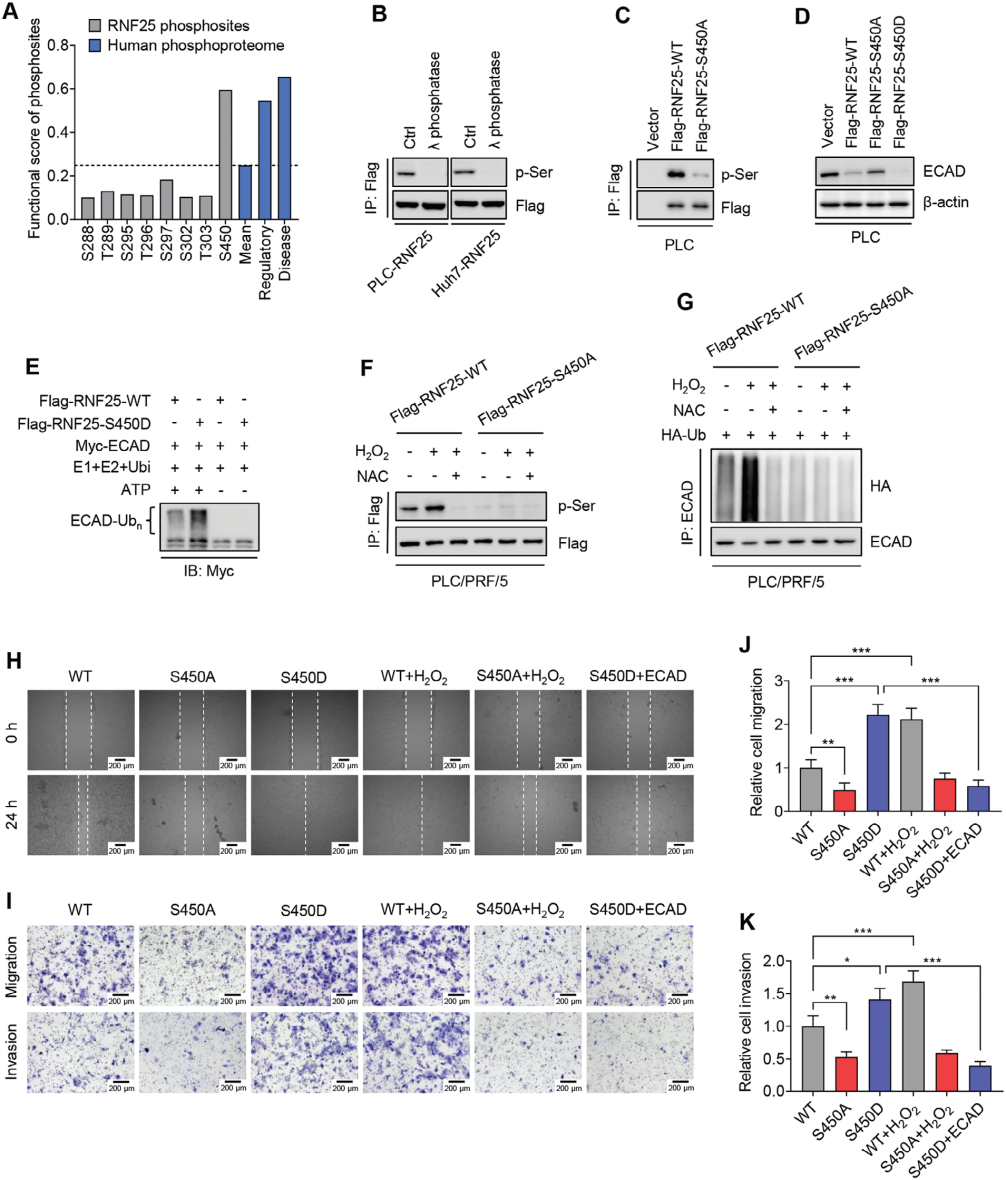
Phosphorylated RNF25 at Ser450 initiates E‐cadherin protein degradation. A) Functional score of phosphosites in RNF25 (gray) and human phosphoproteome (blue) according to a human phosphoproteome dataset. Mean, average score for all human phosphosites. Regulatory, phosphosites with known regulatory functions. Disease, phosphosites related to human diseases. B) Immunoprecipitation assays showing the phosphorylation of RNF25 in HCC cell lysate treated with or without lambda protein phosphatase. C) Immunoprecipitation assays showing the phosphorylation of wild‐type and S450A mutated RNF25 in PLC/PRF/5 stable cells. D) Western blot showing the ECAD protein levels in indicated stable cells. E) In vitro ubiquitination assay showing the ubiquitination of ECAD by wild‐type or S450D mutated RNF25. F) Immunoprecipitation assays showing the phosphorylation of RNF25 in indicated cells with or without the treatment of 100 µm H_2_O_2_ or 1 mm NAC for 1 h. G) Immunoprecipitation assays showing the ubiquitination of ECAD in indicated cells with or without the treatment of 100 µm H_2_O_2_ or 1 mm NAC for 24 h. H) Wound healing assay showing the migration of PLC‐RNF25‐WT, PLC‐RNF25‐S450A, and PLC‐RNF25‐S450D cells with or without the overexpression of ECAD or the treatment of 100 µm H_2_O_2_. Scale bars, 200 µm. I–K) Transwell assay showing the migration and invasion of indicated cells treated as (H). Scale bars, 200 µm. Data are mean ± SD from at least three independent repeats. *P*‐value was determined by an unpaired *t*‐test (J,K). ^*^ *p* < 0.05, ^**^ *p* < 0.01; ^***^ *p* < 0.001.

Since phosphorylation is a common regulatory mechanism for a number of E3 ligases including Nedd4‐2, ITCH, and Smurf1,^[^ ^15^, ^16^, ^17^ ^]^ we wondered if the E3 ligase function of RNF25 is activated by phosphorylation. To this end, a human phosphoproteome dataset was explored,^[^ ^18^ ^]^ which indicated that Ser450 is a potential phosphorylation site of RNF25 with a high functional score (**Figure** **3**A). Phosphorylated RNF25 was detectable in HCC cells, but disappeared when λ protein phosphatase was added. These results indicated that phosphorylation of RNF25 occurred in HCC cells (Figure 3B). To further characterize the phosphorylation site of RNF25 protein, stable PLC and Huh7 cells expressing S450A or S450D mutated RNF25 were generated and used to detect RNF25 phosphorylation. Strong phosphorylation of wild‐type RNF25 was detected. However, the phosphorylation of RNF25 was largely abolished by the S450A mutation of RNF25 (Figure 3C; Figure S3A, Supporting Information). Moreover, the expression of ECAD was partially restored in RNF25 S450A cells but further repressed in RNF25 S450D cells in comparison with RNF25 wild‐type cells (Figure 3D; Figure S3B, Supporting Information). This observation is consistent with an in vitro ubiquitination assay showing an increase of ECAD ubiquitination induced by S450D mutated RNF25 compared with wild‐type RNF25 (Figure 3E). Together, these findings indicate that phosphorylation of RNF25 at Ser450 activated ECAD degradation. Furthermore, the phosphorylation of RNF25 at Ser450 and the consequent ECAD ubiquitination and degradation were dramatically activated in HCC cells after H_2_O_2_ stimulation (Figure 3F,G; Figure S3C,D, Supporting Information). The H_2_O_2_‐induced activation of RNF25 was abolished by treatment with the antioxidant NAC (Figure 3F,G; Figure S3C,D, Supporting Information). All these results suggest that oxidative stress activates RNF25 protein phosphorylation to induce ECAD protein degradation.

To identify the role of oxidative stress‐mediated RNF25 Ser450 phosphorylation in HCC metastasis, the migration and invasion potential of HCC cells with different RNF25 statuses was evaluated after H_2_O_2_ stimulation. S450A mutation of RNF25 significantly abolished the pro‐migratory and pro‐invasive functions of RNF25, while S450D mutation of RNF25 augmented both functions (Figure 3H–K; Figure S3E, Supporting Information). Additionally, H_2_O_2_ treatment further enhanced the migrative and invasive abilities elevated by wild‐type RNF25, but not S450A mutated RNF25 (Figure 3H–K; Figure S3E, Supporting Information). Furthermore, overexpression of exogenous ECAD was shown to inhibit migration and invasion of HCC cells harboring S450D mutated RNF25 (Figure 3H–K; Figure S3E, Supporting Information). Together, these results indicated that ROS‐induced phosphorylation of RNF25 at Ser450 enhances E‐cadherin degradation, leading to elevated migration and invasion potential of HCC cells.

### 2.4 Protein Kinase a Phosphorylates RNF25 at S450

**Figure 4:**
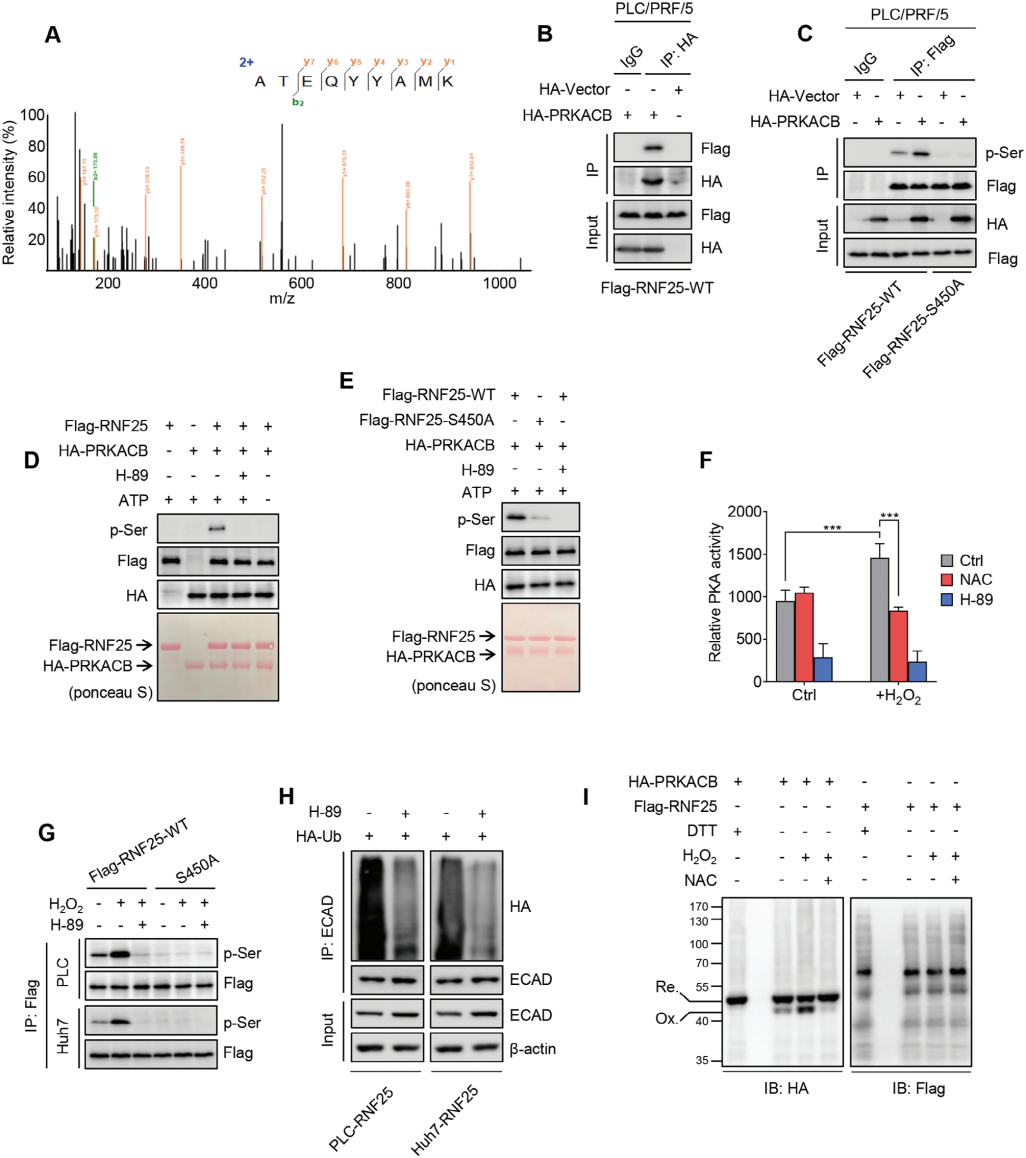
Protein Kinase A phosphorylates RNF25 at S450. A) Spectra of PRKACB obtained from RNF25‐interacted proteins by co‐IP followed by MS assay. B) HA‐tagged PRKACB was transiently expressed in Flag‐RNF25‐WT PLC, followed by detection of the interaction between PRKACB and RNF25 by co‐IP assays. C) Immunoprecipitation assays showing the phosphorylation of wild‐type or S450A mutated RNF25 in PLC cells with or without transient overexpression of PRKACB. D,E) In vitro kinase assays showing the phosphorylation of wild‐type or S450A mutated RNF25 with or without 1 µm PKA inhibitor H‐89. F) PKA activity was determined with or without the treatment of H_2_O_2_ (100 µm), NAC (1 mm), or H‐89 (1 µm) for 1 h. G) Phosphorylation of wild‐type or S450A mutated RNF25 was determined with or without the treatment of H_2_O_2_ (100 µm) or H‐89 (1 µm) for 1 h. H) Immunoprecipitation assays showing the ubiquitination of ECAD in indicated cells treated with or without 1 µm H‐89 for 24 h. I) Non‐reduced SDS‐PAGE showing the band shift due to the formation of an intracellular disulfide bond in PRKACB using HEK293T cells treated with or without 100 µm H_2_O_2_ or 1 mm NAC for 1 h. Ox., oxidized form. Re., reduced form. Data are mean ± SD from at least three independent repeats. *P*‐value was determined by unpaired *t*‐test (F). ^*^ *p* < 0.05, ^**^ *p* < 0.01; ^***^ *p* < 0.001.

To identify the responsible kinase of RNF25 S450 phosphorylation, binding partners of RNF25 were identified through co‐IP followed by MS. Among the top 10 interactors (Table S2, Supporting Information), the catalytic subunit beta of cAMP‐dependent protein kinase (β catalytic subunit of PKA, PRKACB) is the only one annotated with a kinase function (**Figure** **4**A). In general, PKA consists of catalytic and regulatory subunits, the latter keeping PKA in an inactive form.^[^ ^19^ ^]^ Regulatory subunits of PKA are dissociated with catalytic subunits in response to active signals (such as cAMP), thereby rendering catalytic subunits free to phosphorylate their substrates. Interestingly, it has been reported that PKA can also be activated without dissociating the regulatory and catalytic subunits.^[^ ^20^ ^]^ Thus, the physical separation of the regulatory subunits might not be a prerequisite for the activation of catalytic subunits. Interestingly, we found that PRKACB interacts with RNF25 protein and promotes the phosphorylation of RNF25 at the S450 site in HCC cells (Figure 4B,C; Figure S4A,B, Supporting Information). In addition, in vitro kinase assays showed that PRKACB induces phosphorylation of RNF25, and the PRKACB‐activated phosphorylation of RNF25 was undetectable when RNF25 was mutated at the S450A (Figure 4D,E). These observations indicate that PRKACB can phosphorylate RNF25 at S450.

It is well known that the activity of PKA is, like other kinds of kinase, regulated by oxidative stress.^[^ ^21^ ^]^ We therefore explored whether PRKACB responds to oxidative stress. As shown, ROS stimulated the activity of PRKACB, which can be inhibited by NAC treatment (Figure 4F). Moreover, H_2_O_2_ or TGFβ1‐induced phosphorylation of RNF25 at S450 was inhibited by treatment with H‐89 (a PKA inhibitor) or NAC (Figure 4G; Figure S4C, Supporting Information). Consistently, inhibition of PKA by H‐89 decreased the ubiquitination of ECAD in HCC cells and impeded the pro‐invasive effect of H_2_O_2_ in HCC cells (Figure 4H; Figure S4D, Supporting Information). These results indicate that the β subunit of PKA is a sensor of ROS to mediate RNF25 phosphorylation in response.

To further confirm if PKA is a redox sensor, non‐reducing SDS PAGE assays were performed to identify whether PKA is inducible by ROS. A band shift of PRKACB, but not RNF25 protein was detected after H_2_O_2_ treatment. The shift of PRKACB was inhibited by NAC treatment (Figure 4I). These results indicate that PRKACB, but not RNF25, is a typical redox sensor that directly senses ROS through the formation of an intramolecular disulfide bond.

### 2.5 Redox Modification of PRKACB at Cys200/344 Activates PKA Kinase Activity

**Figure 5:**
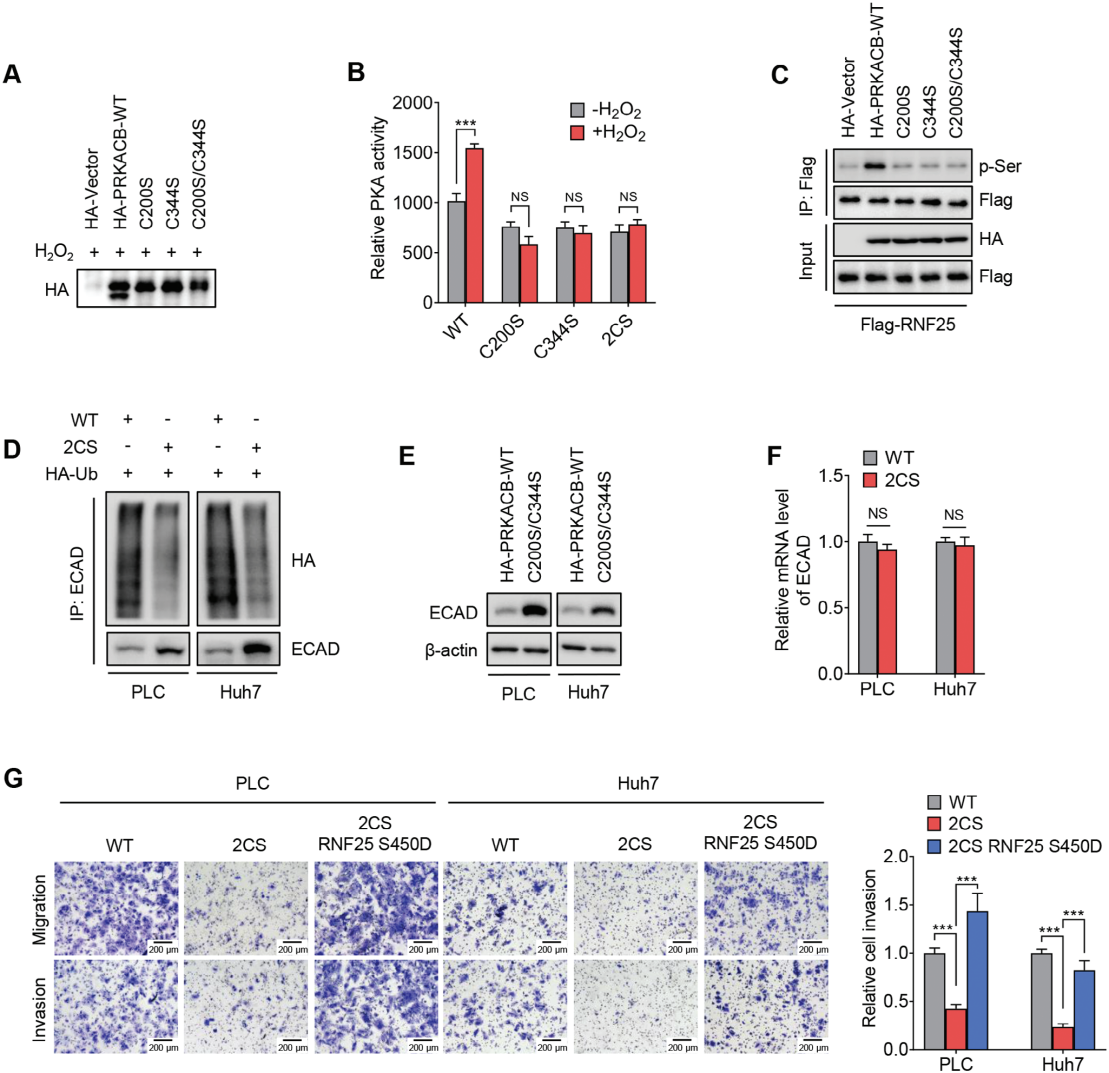
Redox modification of Protein Kinase A at C200/344 upregulates kinase activity of PKA. A) The band shift was detected in non‐reduced SDS‐PAGE using HEK293T cells expressing wild‐type or cysteine‐mutated PRKACB. The cells were treated with 100 µm H_2_O_2_ for 1 h before harvesting. B) Activity of wild‐type or cysteine mutated PRKACB was measured with or without the treatment of 100 µm H_2_O_2_ for 1 h. C) The phosphorylation of RNF25 by wild‐type or cysteine‐mutated PRKACB was determined with immunoprecipitation assays using HEK293T cells. D) Co‐IP assay showing the ubiquitination of ECAD in HCC cells expressing wild‐type or C200S/C344S mutated PRKACB. E,F) Immunoblotting and qPCR assays showing the protein expression and mRNA levels of ECAD in HCC cells expressing wild‐type or C200S/C344S mutated PRKACB. G) Transwell assay showing the migration and invasion of HCC cells with or without the mutation of PRKACB and the ectopic expression of S450D RNF25. Data are mean ± SD from at least three independent repeats. *P*‐value was determined by unpaired *t*‐test (B,F,G). ^*^ *p* < 0.05, ^**^ *p* < 0.01; ^***^ *p* < 0.001. NS, not significant.

Interestingly, there are only two cysteines within the human PRKACB protein, namely C200 and C344.^[^ ^22^ ^]^ As a result, mutation of either cysteine or both abolished the formation of an intramolecular disulfide bond (**Figure** **5**A). Moreover, the mutation of cysteines of PRKACB abolished the H_2_O_2_‐induced PRKACB kinase activation (Figure 5B) and subsequent RNF25 protein phosphorylation (Figure 5C). Besides, the protein expression of ECAD was upregulated due to the ubiquitination of ECAD protein was not induced in cells with C200/344 mutant PRKACB (Figure 5D–F). Consistently, cellular migration and invasion were inhibited in HCC cells with C200/344 mutant PRKACB. Mutant PRKACB‐induced inhibition of migration and invasion was overcome by exogenous expression of S450D mutant RNF25 (Figure 5G). Together, this data indicates that oxidation of C200/344 of PRKACB enhances PKA kinase activity to enable RNF25 phosphorylation and ECAD degradation, ECAD degradation subsequently induces migration and invasion of HCC cells.

### 2.6 ROS‐Induced RNF25 Phosphorylation Degrades ECAD Protein to Promote HCC Metastasis

To further confirm the critical role of the ROS‐PKA‐RNF25‐ECAD axis in HCC metastasis, we first investigated the role of RNF25 in HCC metastasis in vitro. The migration and invasion of HCC cells were evaluated in HCC cells with different RNF25 statuses. The overexpression of RNF25 was found to enhance cell mobility. Consistently, cellular migration and invasion were inhibited in cells with RNF25 silencing (Figure S5A–E, Supporting Information). Furthermore, RNF25‐induced cellular migration and invasion were augmented in H_2_O_2_‐stimulated HCC cells and overcome in HCC cells with exogenous ECAD expression (Figure S5A–D, Supporting Information). In addition, RNF25‐induced and H_2_O_2_ stimulation augmented cellular migration and invasion was undetectable after antioxidant NAC treatment in HCC cells. By contrast, overexpression or knockdown of RNF25 did not significantly affect the proliferation of HCC cells (Figure S5F–I, Supporting Information). These results indicate that ROS‐regulated cell migration and invasion are mediated by RNF25‐induced ECAD protein degradation.

**Figure 6:**
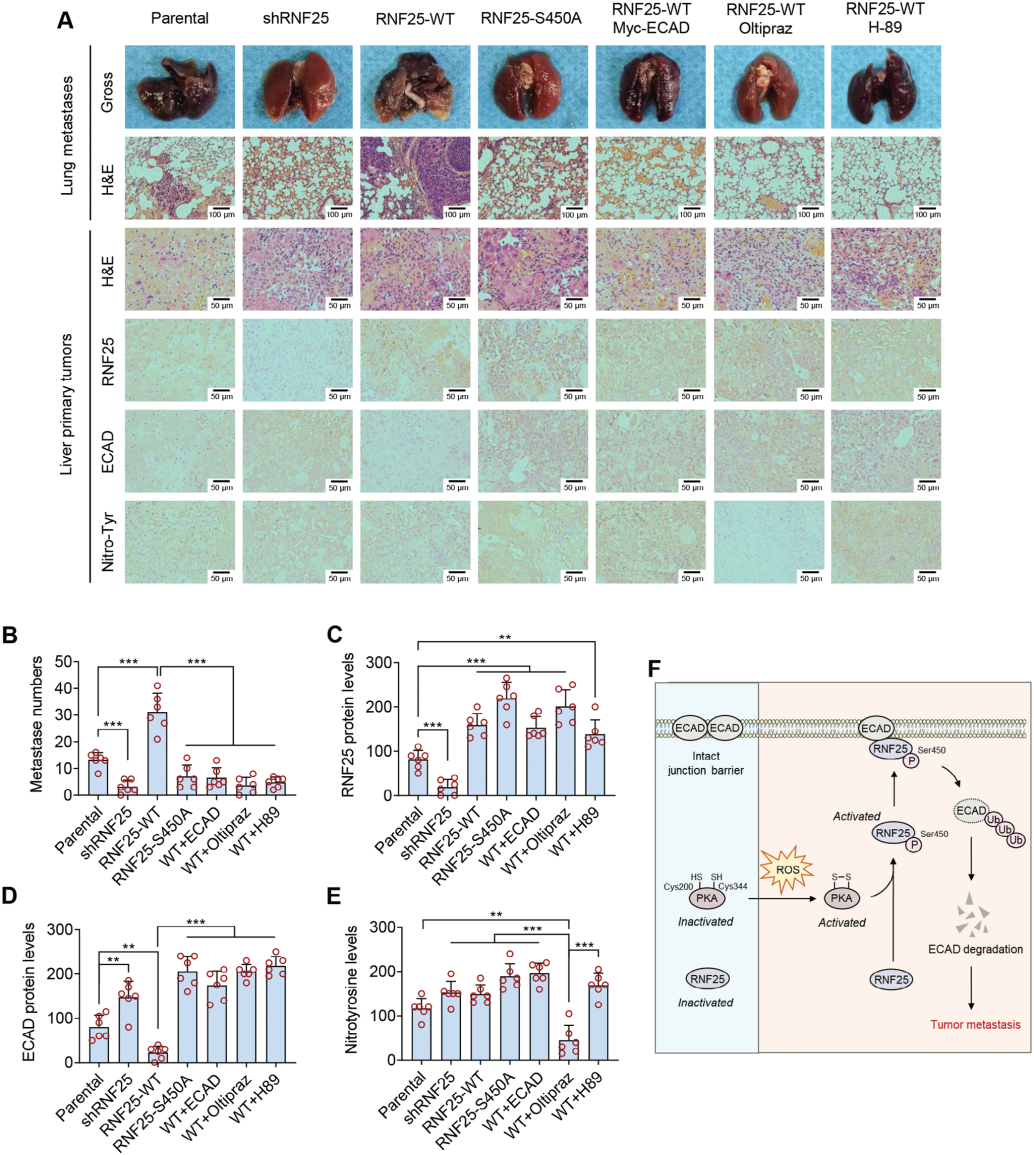
ROS‐induced RNF25 phosphorylation degrades ECAD protein to promote HCC metastasis. A) Evaluation of liver cancer lung metastasis by an orthotopic mouse model using Huh7 cells with or without RNF25 knockdown, RNF25 overexpression, RNF25 S450A mutation, ECAD rescue, oltipraz (Nrf2 agonist) treatment or H‐89 (PKA inhibitor) treatment. The histology of lung metastases and liver primary tumors was analyzed by H&E staining, and the protein levels of RNF25, ECAD, and nitrotyrosine were determined by immunohistochemistry (IHC). Among them, nitrotyrosine was used as the indicator for oxidative stress. B–E) Quantifications for each group in (A). Scale bars, 100 µm (lung) or 50 µm (liver). F) Working model of ROS‐mediated HCC metastasis regulated by PKA‐RNF25‐ECAD axis. In response to ROS, PRKACB forms a disulfide bond between Cys200 and Cys344, resulting in an increase in PKA kinase activity. Then, PKA phosphorylates RNF25 at Ser450, enhancing its E3 activity. This event leads to the ubiquitination and subsequent protein degradation of ECAD, thereby facilitating metastasis. Data are mean ± SD from six mice in each group. *P*‐value was determined by unpaired *t*‐test (B–E). ^*^ *p* < 0.05, ^**^ *p* < 0.01; ^***^ *p* < 0.001.

To identify the role of ROS‐PKA‐RNF25‐ECAD axis in vivo, an orthotopic mouse model was generated using HCC cells expressing wild‐type or S450A mutated RNF25 followed by different treatments. RNF25 silencing significantly repressed the formation of lung metastases, while overexpression of exogenous RNF25 dramatically enhanced metastasis (**Figure** **6**A,B). In contrast, overexpression of S450A‐mutated RNF25 failed to facilitate metastasis (Figure 6A,B). The rescue of ECAD expression also decreased the pro‐metastatic effect of RNF25. This result confirmed that RNF25‐promoted HCC metastasis is mediated by the downregulation of ECAD (Figure 6A,B). Moreover, the administration of oltipraz, an agonist of the central antioxidant gene Nrf2, was shown to upregulate RNF25 to inhibit metastasis (Figure 6A,B). Furthermore, treatment with PKA inhibitor H‐89 can also suppress RNF25‐induced HCC metastasis (Figure 6A–C). Immunohistochemistry (IHC) was also performed to confirm the correlation between the expression of RNF25 and ECAD and HCC metastasis (Figure 6A–E). By contrast, the primary tumor size was comparable in each group, suggesting that the PKA/RNF25/ECAD pathway is mainly involved in the regulation of tumor metastasis rather than tumor growth (Figure S6, Supporting Information). Together, these findings suggest the critical role of the ROS‐PKA‐RNF25‐ECAD axis in HCC metastasis in vivo (Figure 6F).

**Figure 7:**
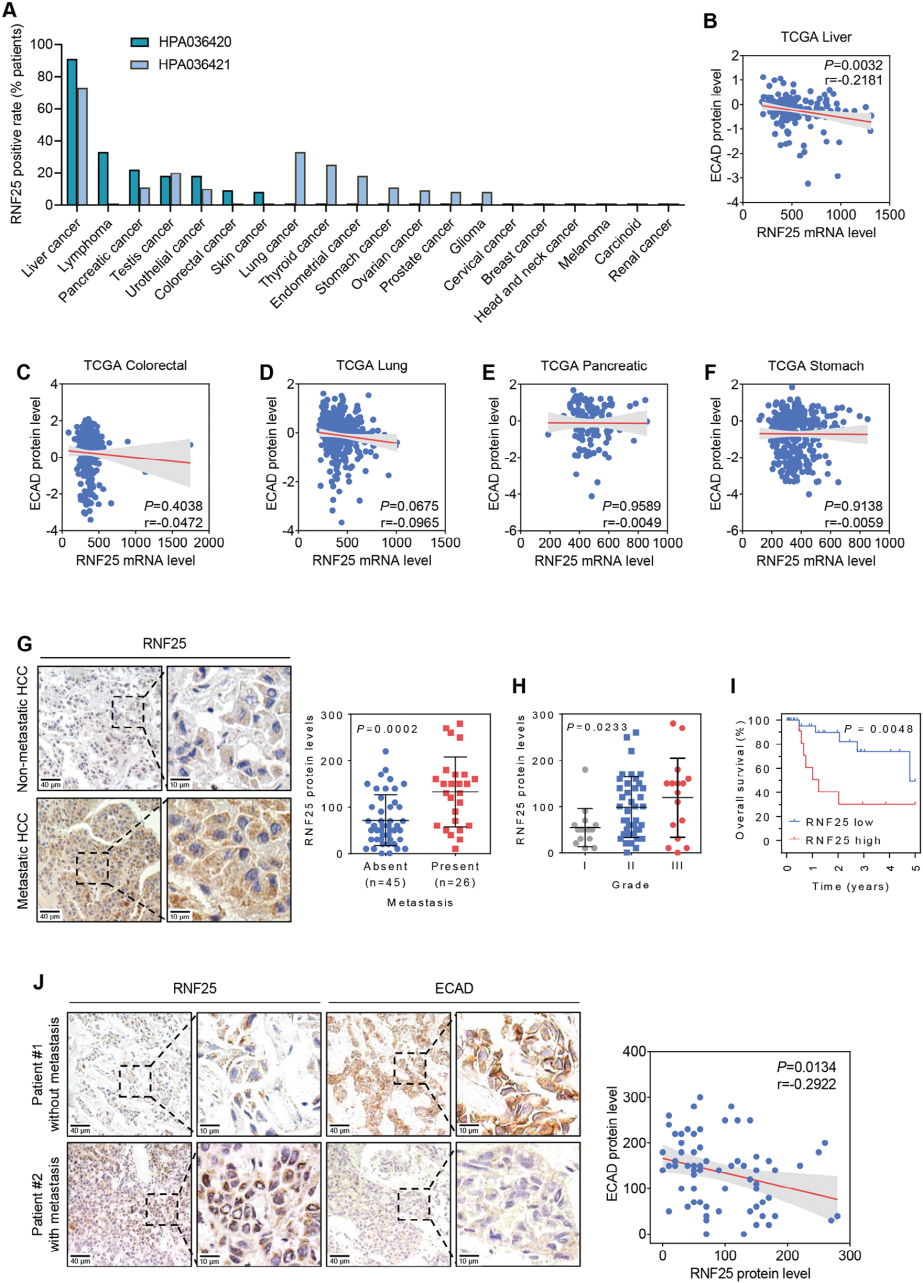
RNF25 is overexpressed in human HCC tissue and associated with poor prognosis. A) RNF25 protein levels in patients with indicated cancer types according to The Human Protein Atlas dataset. HPA036420 and HPA036421 represent two distinct antibodies recognizing RNF25 protein. B–F) The correlation between RNF25 mRNA level and ECAD protein level in different cancer types was evaluated using the TCGA dataset. G) Immunohistochemistry (IHC) assays showing the protein level of RNF25 in HCC patients with or without metastasis. Scale bars, 40 µm (zoom out) or 10 µm (zoom in). H) HCC patients in (G) were grouped according to their tumor grade followed by evaluation of RNF25 expression. I) HCC patients in (G) were grouped according to their RNF25 expression level followed by an analysis of their overall survival. J) The correlation of protein levels of RNF25 and ECAD in clinical HCC samples was determined by IHC. Scale bars, 40 µm (zoom out) or 10 µm (zoom in). *p*‐value was determined by Pearson correlation test (B–F,J), unpaired *t*‐test (G), one‐way ANOVA (H), and Log‐rank (Mantel‐Cox) test (I). ^*^ *p* < 0.05, ^**^ *p* < 0.01; ^***^ *p* < 0.001.

To identify the histological status of RNF25 in human HCC, The Human Protein Atlas database was used for a pan‐cancer analysis of RNF25. As shown, RNF25 is highly overexpressed in liver cancer specifically, but not in other kinds of tumors (**Figure** **7**A). The expression of RNF25 protein is negatively correlated with the protein level of ECAD in liver cancer of TCGA collected (Figure 7B–F; Figure S7A–D, Supporting Information). This result suggests that the correlation between RNF25 and ECAD protein might be a tissue‐specific event, or at least might more frequently occur in liver cancer. To further investigate the role of the RNF25‐ECAD axis in HCC, the expression of RNF25 protein was analyzed by immunohistochemistry (IHC) in 71 HCC clinical samples. RNF25 protein expression was upregulated in metastatic HCC tissues compared with non‐metastatic samples (Figure 7G). Additionally, RNF25 was enriched in advanced tumors. This result indicates that the expression of RNF25 is associated with tumor progression (Figure 7H). Moreover, Kaplan–Meier analysis demonstrated a poor overall survival of HCC patients with high RNF25 expression (Figure 7I). A negative correlation between the protein level of RNF25 and ECAD was also found in these HCC clinical samples (Figure 7J). Together, these data indicate that high expression of RNF25 is associated with HCC metastasis and poor prognosis.

## 3 Discussion

Oxidative stress drives multiple aggressive behaviors in tumors. However, compelling evidence suggests that ROS affects cancer metastasis in contradictory manners. For those tumor cells that have already entered the blood, oxidative stress inhibits their metastasis by inducing ferroptosis.^[^ ^23^, ^24^ ^]^ Nevertheless, how oxidative stress promotes metastasis at the beginning of metastatic spread, up to now, has been largely unknown. Here, we show that oxidative stress occasionally induces ECAD protein degradation by activating RNF25, leading to EMT in HCC cells. Mechanistic studies revealed that PRKACB, the catalytic subunit of PKA, undergoes redox modification on C200 and C344 in response to oxidative stress, thus leading to an increase in kinase activity. Activated PKA then phosphorylates RNF25 at S450, which facilitates RNF25‐mediated ECAD degradation. Thus, our study reveals a novel mechanism of oxidative stress‐induced tumor metastasis in HCC.

Loss of ECAD has long been recognized as one of the most important early events during cancer metastasis. Though epigenetic silencing of ECAD has been widely reported, relatively few studies have focused on the regulation of ECAD protein levels. In fact, in addition to transcriptional regulation, protein degradation of ECAD has emerged as an alternative mechanism leading to ECAD loss during cancer metastasis. For instance, the glycoprotein CD147 was shown to recruit E3 ligase CBLL1 for ECAD degradation, resulting in tumor progression.^[^ ^25^ ^]^ Such evidence suggests that loss of ECAD can be induced at both the mRNA and protein levels, but which pathway is preferential at a given circumstance remains to be clarified. Here, our findings address the importance of a protein degradation pathway as a critical regulatory mechanism in stress management. Based on our findings, we can speculate that in RNF25‐high HCC cells, ECAD can be repressed by ubiquitin‐mediated protein degradation in response to oxidative stress. By contrast, in RNF25‐low HCC cells, the common epigenetic silencing may be the dominant mechanism leading to ECAD downregulation. This view is supported by the literature which describes that ROS can stimulate the nuclear translocation of EMT‐related transcription factors such as Snail, which is a well‐known ECAD repressor, by epigenetic silencing.^[^ ^26^ ^]^ The basal level (both mRNA and protein) of ECAD is low in SK‐Hep‐1 cells, suggesting constitutive epigenetic repression of ECAD even in the absence of ROS, but this speculation needs further investigation.

In addition, our findings also suggest a dynamic regulation of redox signaling and ECAD expression in the different stages of tumor metastasis. β‐catenin is a binding partner of ECAD on the cell membrane. When ECAD is downregulated, β‐catenin can translocate into the nucleus thereby activating Wnt target genes.^[^ ^27^ ^]^ Wnt signaling is an important stemness‐related pathway that contributes to the formation of cancer stem cells (CSCs).^[^ ^28^ ^]^ CSCs have powerful antioxidant capacity conferring survival under oxidative stress, which facilitates tumor metastasis.^[^ ^29^ ^]^ Therefore, based on our findings and published literature, we speculated that the interplay between RNF25, ECAD, and β‐catenin promotes tumor dissemination in different metastatic stages. At the early stage, ROS downregulates ECAD via RNF25‐mediated ECAD protein degradation, leading to EMT of tumor cells. At the advanced stage, β‐catenin activates Wnt signaling to enhance the antioxidant capability of tumor cells and enable ECAD re‐expression and subsequent distant colonization. These metastatic processes are also featured by mitochondria‐related metabolic reprogramming,^[^ ^30^, ^31^ ^]^ while mitochondria are the key sources of intracellular ROS.

Inactive PKA is a holoenzyme consisting of catalytic and regulatory subunits. In the presence of cAMP, the regulatory subunits are physically separated from catalytic subunits, leading to PKA activation.^[^ ^19^ ^]^ Though not conventionally considered an oncogene, compelling evidence suggests that PKA is involved in cancer development, which is at least partially due to its function of coping with stress. For instance, activation of PKA promotes the survival of drug‐resistant cancer cells in stressful conditions, including nutrient deprivation and cell detachment.^[^ ^32^ ^]^ Besides, tumor growth can be accelerated in stressed animal models by activating PKA.^[^ ^33^ ^]^ In line with this, changes in the level of cAMP, the agonist of PKA, significantly alters stress‐induced behavior in mice.^[^ ^34^ ^]^ As one of the most common stress conditions, oxidative stress also leads to the activation of PKA.^[^ ^35^ ^]^ Interestingly, in response to oxidative stress, PKA was found to form an intermolecular disulfide bond between its two regulatory subunits, leading to its activation independent of cAMP.^[^ ^36^ ^]^ However, whether its catalytic subunits undergo redox modifications remains undetermined. Here, we show that PRKACB directly senses oxidative stress by the formation of an intramolecular disulfide bond between C200 and C344, which increases its kinase activity, leading to the phosphorylation of RNF25 and consequent ECAD degradation in HCC. This finding demonstrates that PRKACB is a typical redox sensor. Another catalytic subunit, PRKACA, shares more than 90% similarity in amino acid sequence with PRKACB including cysteines C200 and C344, suggesting that a similar redox regulation might also exist in PRKACA, which deserves further investigation.

Surgery is the crucial therapeutic option for patients with the most solid tumors that controls cancer progression and prolongs patient survival worldwide. However, it has been reported that surgical resection unexpectedly accelerates tumor recurrence in some cases.^[^ ^37^, ^38^ ^]^ This paradox is thought to be attributed to the multifaceted inflammatory response induced by traumatic stress. Pro‐inflammatory factors are required to activate the immune system for the elimination of cancer cells, but an inflammatory tumor microenvironment frequently supports the development of cancer. Surgical procedures occasionally stimulate oxidative stress, which facilitates tumor growth and metastasis.^[^ ^39^ ^]^ In fact, surgery induces an increased level of inflammation, where proinflammatory cytokines can stimulate the production of ROS. Thus, modulation of ROS levels (such as administration of neutralizing antibodies against TGFβ and IL‐6) in the postoperative period may improve the clinical outcomes of patients undergoing surgery.

In summary, our study indicates that oxidative stress mediates a redox modification on the β subunit of protein kinase A, which occasionally drives tumor metastasis via the RNF25‐mediated ECAD protein degradation in HCC. This finding helps explain how oxidative stress‐induced ECAD degradation promotes tumor metastasis in HCC.

## 4 Experimental Section

### Antibodies and Reagents

Antibodies for E‐cadherin (Cell Signaling Technology, Cat # 3195), ZO‐1 (Cell Signaling Technology, Cat # 8193), N‐cadherin (Cell Signaling Technology, Cat # 13 116), Vimentin (Cell Signaling Technology, Cat # 5741), Acetylated‐Lysine (Cell Signaling Technology, Cat # 9441), Ubiquitin (Abcam, Cat # ab134953), β‐actin (Santa Cruz Biotechnology, Cat # sc‐69879), RNF25 (Abcam, Cat # ab89281), HA‐tag (Abcam, Cat # ab49969), Myc‐tag (Cell Signaling Technology, Cat # 2278), Flag‐tag (Cell Signaling Technology, Cat # 14 793), Nitrotyrosine (Merck, Cat # 05–233), p‐Serine (ZEN‐Bioscience, Cat # 530 893), and reagents including N‐acetyl cysteine (NAC) (Merck, Cat # A9165), Recombinant Human TGF‐β1 (PEPROTECH, Cat # 100–21), Recombinant Human IL‐6 (PEPROTECH, Cat # 200–06), Recombinant Human EGF (MedChemExpress, Cat # HY‐P7109), Hydrogen peroxide solution (H_2_O_2_) (Merck, Cat # 323 381), Lambda Protein Phosphatase (Beyotime Biotechnology, Cat # P2316S), MG132 (Aladdin, Cat # M126521), Oltipraz (Aladdin, Cat # O136690), H‐89 (Aladdin, Cat # H129712), Kemptide (Aladdin, Cat # K118853) were purchased commercially.

### Cell Culture

Liver cancer cell lines PLC/PRF/5, Huh7, HLE, HepG2, HLF, SK‐Hep‐1, and the embryonic kidney cell line HEK293T were cultured in our lab and maintained in Dulbecco's modified Eagle's medium (DMEM, Gibco) containing fetal bovine serum (10%, BI), penicillin (100 U mL^−1^, Invitrogen) and streptomycin (100 µg mL^−1^, Invitrogen) at 37 °C with 5% CO_2_. To generate RNF25 stable knockdown and overexpression cells, lentivirus harboring shRNA (sh#1: 5′‐GATGAACTACAGGTGATTAAA‐3′ and sh#2: 5′‐ GTTCGCTGGGAGCGCTCTAAA‐3′) or cDNA for RNF25 was used to infect HCC cells followed by puromycin selection. The RNF25 S450A and S450D stable cells were obtained through similar approaches based on shRNF25 #1 stable cells.

### Animal Models

Animal studies were approved by the Institutional Animal Care and Treatment Committee of Sichuan University. Female BALB/c nude mice (6‐week‐old, HFK Bioscience) were raised in the SPF Laboratory Animal Center of Sichuan University. For the orthotopic model in Figure 1, 1×10^6^ Huh7 cells were suspended at 20 µL PBS and injected into the left liver lobe of mice. One week post injection, mice were treated with or without NAC (50 mg kg^−1^ per day, i.p.) for 2 weeks. Then, mice were kept without drug administration for another 3 weeks. For the orthotopic model in Figure 6, 1 × 10^6^ parental, shRNF25, Flag‐RNF25‐WT, Flag‐RNF25‐S450A, Flag‐RNF25‐WT+Myc‐ECAD PLC/PRF/5 cells were suspended at 20 µL PBS and injected into the left liver lobe of mice with or without the treatment of Oltipraz (10 mg kg^−1^ per day, p.o.) or H‐89 (0.5 mg kg^−1^ per day, i.p.) for 2 weeks. For each group *n* = 6. One month post injection, mice were euthanized. The lungs were collected for photographic examination and the metastases were counted. Then, the lungs and livers were fixed in 4% formaldehyde for histological analysis.

### Clinical HCC Tissues and IHC

Studies using clinical samples were approved by the Biomedical Ethics Review Committee of Sichuan University. Human HCC samples (cohort 1, *n* = 42 in Figure 1 and cohort 2, *n* = 71 in Figure 7) were collected from West China Hospital, Sichuan University, and grouped according to their pathological characteristics including metastasis status, cirrhosis status, and tumor grade as shown in the medical records. IHC assays were performed as previously described.^[^ ^40^ ^]^ Briefly, human or mouse HCC tissues were embedded in paraffin and then sliced into 5 µm thickness sections for preservation. To detect protein expression, the sections were rehydrated and treated with 3% H_2_O_2_ for 15 min to block endogenous peroxidase. Next, the slides were incubated with antigen retrieval buffer and then boiled for 4 min. After blocking with goat serum, sections were treated with primary antibodies at 4 °C overnight. After washing three times with phosphate‐buffered saline (PBS), the sections were incubated with MaxVision HRP solution (MXB Biotechnology, Cat # 5020) reagent for 1 h at 25 °C. Next, the slides were washed again three times with PBS, followed by staining using DAB Peroxidase Substrate (MXB Biotechnology, Cat # 0031). Nuclei were stained with hematoxylin. Photographs were captured using a DM2500 fluorescence microscope (Leica).

### Transwell Assay

Polycarbonate membrane inserts with an 8 µm pore (Corning, cat # 3422) were used in the Transwell assays, the experiment being performed as previously described.^[^ ^40^ ^]^ Briefly, to detect the migration of cells, 600 µL growth medium was added in 24‐well plates prior to placing the insert into the well. Then, 1 × 10^5^ cells were resuspended with 200 µL FBS‐free medium followed by seeding into the chamber. To measure the invasion of cells, the chamber was pre‐coated with matrigel (BD Biosciences, Cat # 356 234). Next, 2 × 10^5^ cells were resuspended in 200 µL FBS‐free medium and seeded into the insert. The plate containing insets was then cultured at 37 °C in a humidified chamber for 24 h. After that, migrated or invaded cells were fixed with 4% formaldehyde for 30 min at room temperature followed by staining with 0.1% crystal violet for another 30 min at room temperature. Photographs were captured using a DM2500 fluorescence microscope (Leica).

### Co‐Immunoprecipitation

The co‐IP assays were performed as previously described.^[^ ^40^ ^]^ Briefly, 3 × 10^6^ cells were lysed with NP‐40 lysis buffer (0.5%, pH 7.4) containing 20 mm Tris, 100 mm NaCl, and 0.5 mm EDTA supplemented with 1% protease inhibitor and phosphatase inhibitor (Bimake, Cat # B14001, B15001) for 30 min at 4 °C, followed by incubation with 1 µg primary antibodies at 4 °C overnight. Then, cell lysate was incubated with protein G plus protein A agarose beads (Merck, Cat # IP10‐10MLCN) for 2 h at 4 °C. Next, the beads were washed three times using NP‐40 wash buffer with similar constituents as in the NP‐40 lysis buffer except for NaCl (150 mm). The beads were then boiled with SDS‐PAGE loading buffer for 10 min and subjected to immunoblotting analysis.

### Immunoblotting

Cells were washed with ice‐cold PBS twice and then lysed with RIPA lysis buffer (50 mm Tris, 150 mm NaCl, 0.5 mm EDTA, 1% Triton X‐100, 1% sodium deoxycholate, 0.1% SDS, pH 7.4) supplemented with protease inhibitor and phosphatase inhibitor (as above). Next, the lysate was mixed with loading buffer and boiled for 10 min. For non‐reduced SDS‐PAGE to detect the band shift, a non‐reduced loading buffer (DTT‐free) was used to protect the disulfide bond. Next, the proteins were separated by SDS‐PAGE followed by transfer to the PVDF membrane (Merck, Cat # ISEQ00010). Then, the membrane was blocked by TBST buffer containing 5% non‐fat milk powder for 1 h at room temperature, followed by cutting into strips. The strips were then incubated with indicated antibodies at 4 °C overnight, followed by three washings with TBST, and incubated with secondary antibodies for 2 h at room temperature. After six washes with TBST, the strips were incubated with Immobilon Western HRP Substrate (Merck, Cat # WBKLS0500) and the images were obtained using a ChemiScope 6000 Touch Chemiluminescence imaging system (Clinx, Shanghai).

### Quantitative RT‐PCR

Cells were washed with ice‐cold PBS twice and the total RNA was extracted with TRIzol reagent (Thermo Fisher Scientific, Cat # 15 596 018). Then, the RNA was reverse transcribed by the PrimeScript RT reagent Kit (Takara, Cat # RR047A) to yield the cDNA. Next, the PCR reaction was performed using SYBR Green reagent (Bio‐Rad, 1 725 271), and the relative cDNA levels of targeted genes were normalized to β‐actin. The primer sequences are provided as follows (5′ to 3′). E‐cadherin: CAGCACGTACACAGCCCTAA and ACCTGAGGCTTTGGATTCCT. RNF25: ATCTTACAGGTGCTGGGCCA and AACCATAGAGGCAGATGACACAC. ACTB: GACCTGACTGACTACCTCATGAAGAT and GTCACACTTCATGATGGAGTTGAAGG.

### Cell Viability Assays

For MTT assays, cells were seeded in 96‐well plates at a density of 1000 cells per well and cultured at 37 °C overnight. Next, at indicated time points (0, 24, 48, and 72 h), cells were treated with MTT reagent at 37 °C for 3 h, resolved in DMSO and the absorbance detected at 570 nm using a spectrophotometer. The absorbance at 24, 48, and 72 h were normalized to that at the time. For colony formation assays, cells were seeded in 24‐well plates at a density of 500 cells per well and cultured at 37 °C for 2 weeks. Next, cells were washed twice with PBS and fixed with 4% paraformaldehyde for 1 h at room temperature. Then, cells were stained using 0.2% crystal violet for 1 h at room temperature, followed by three washes with PBS. Cells were then photographed, and the colonies were counted.

### Co‐IP Followed by Mass Spectrometry (MS)

Cells expressing Myc‐tagged ECAD or Flag‐tagged RNF25 were lysed with IP lysis buffer supplemented with protease inhibitor and phosphatase inhibitor. Then, cell lysates were incubated with Anti‐Myc‐tag or anti‐Flag‐tag agarose at 4 °C overnight, followed by three washes with IP lysis buffer and centrifugation at 2000 × g for 3 min and boiled for 10 min. Next, the immunoprecipitated proteins were subjected to SDS‐PAGE, and the bands of interest were cut into pieces. Then, the samples were subjected to MS analysis by Shanghai Applied Protein Technology Co., Ltd. Briefly, LC‐MS/MS analysis was performed on a Q Exactive mass spectrometer (Thermo Scientific) that was coupled to Easy nLC (Proxeon Biosystems, now Thermo Fisher Scientific) for 60 min. The mass spectrometer was operated in positive ion mode. MS data was acquired using a data‐dependent top20 method dynamically choosing the most abundant precursor ions from the survey scan (300–1800 m z^−1^) for HCD fragmentation. The automatic gain control (AGC) target was set to 1e6, the maximum injects time to 50 ms, and the number of scan ranges to 1. Dynamic exclusion duration was 30.0 s. Survey scans were acquired at a resolution of 70000 at m z^−1^ 100 and the resolution for HCD spectra was set to 17500 at m z^−1^ 100, The Automatic gain control (AGC) target was set to 1e5, and the isolation width was 1.5 m z^−1^, micro scans to 1, and maximum inject time to 50 ms. The normalized collision energy was 27 eV and the underfill ratio, which specifies the minimum percentage of the target value likely to be reached at maximum fill time, was defined as 0.1%. The instrument was run with peptide recognition mode enabled. MS/MS spectra were searched using MASCOT (Matrix Science, London, UK; version 2.2) against a nonredundant International Protein Index Arabidopsis sequence database v3.85 (released in September 2011; 39 679 sequences) from the European Bioinformatics Institute (http://www.ebi.ac.uk/).

### PKA Protein Kinase Activity Assay

Wild type, C200S mutated, C344S mutated or double mutated HA‐tagged PRKACB were overexpressed in cells and immunoprecipitated by anti‐HA tag antibody together with agarose beads as described above. Then, the beads were eluted with an equal volume of glycine butter (0.2 m glycine, pH 2.6) for 10 min and neutralized using an equal volume of pH 8.0 Tris buffer. Next, purified proteins were subjected to an activity assay with Kemptide (Aladdin, Cat # K118853) as the exogenous substrate at 30 °C for 10 min. The reaction mixture consists of 10 µg Kemptide, 1 µg PRKACB, 10 mm MgCl_2_, 20 µm ATP, and 50 mm Tris‐HCl pH 7.4 in a total volume of 100 µL. Enzymatic activity was determined by the consumption of ATP, which was measured using an ATP assay kit (Beyotime, Cat # S0026) according to the manufacturer's instructions.

### In Vitro Ubiquitination Assay

His‐tagged ubiquitin, His‐tagged E1 (UBA1), His‐tagged E2 (UBE2D2), Flag‐tagged E3 (RNF25‐WT or RNF25‐S450D), Myc‐tagged substrate (E‐cadherin) were overexpressed in cells and purified as above. Then, the assay was performed using a ubiquitination buffer (10 µm ubiquitin, 10 nm E1, 100 nm E2, 100 nm E3, 500 nm substrate, 5 mm ATP, 5 mm MgCl_2_, 1 mm DTT, 50 mm Tris‐HCl, pH 7.4) in a total volume of 50 µL. The reaction proceeded at 30 °C for 2 h, followed by the addition of SDS‐PAGE loading buffer and boiling for 10 min. The samples were then subjected to Western blot for the measurement of ubiquitination.

### In Vitro Phosphorylation Assay

HA‐tagged kinase (PRKACB) and Flag‐tagged substrate (RNF25‐WT or RNF25‐S450A) were overexpressed in cells and purified as above. Then, the assay was performed using a kinase buffer (0.1 µm kinase, 1 µm substrate, 150 mm NaCl, 10 mm MgCl_2_, 1 mm DTT, 0.1 mm ATP, 50 mm Hepes/KOH, pH 7.5) in a total volume of 50 µL. The reaction proceeded at 30 °C for 20 min, followed by the addition of SDS‐PAGE loading buffer and boiling for 10 min. The samples were subjected to Western blot for the measurement of phosphorylation.

### Statistical Analysis

Statistical analysis was performed using GraphPad Prism software (version 8.3.0). Data were presented as mean ± SD obtained from at least three independent assays unless otherwise indicated. Data in different groups were analyzed by two‐tailed Student's *t*‐test or one‐way ANOVA. The correlation was evaluated by Pearson correlation test. Kaplan–Meier analysis for patient survival was determined by log‐rank (Mantel–Cox) test. Other statistical methods are shown in figure legends. *P* values < 0.05 were considered statistically significant.

## Conflict of Interest

The authors declare no conflict of interest.

## Supporting information

Supporting Information

## Data Availability

The data that support the findings of this study are available from the corresponding author upon reasonable request.
