# Supplementary material for: Oxidative Stress Promotes Liver Cancer Metastasis via RNF25‐Mediated E‐Cadherin Protein Degradation
Source: Adv Sci (Weinh). 2024 Jan 29;11(13):2306929. doi: 10.1002/advs.202306929 (PMC10987140; doi:10.1002/advs.202306929)
Supplement: Supplementary file 1 — Supporting Information [file ADVS-11-2306929-s001.pdf]

ADVANCED SCIENCE
Open Access

Supporting Information

for *Adv. Sci.*, DOI 10.1002/advs.202306929

Oxidative Stress Promotes Liver Cancer Metastasis via RNF25-Mediated E-Cadherin Protein Degradation

Zhao Huang, Li Zhou, Jiufei Duan, Siyuan Qin, Jingwen Jiang, Haining Chen, Kui Wang, Rui Liu, Minlan Yuan, Xiangdong Tang, Edouard C. Nice, Yuquan Wei, Wei Zhang and Canhua Huang**

Oxidative stress promotes liver cancer metastasis via RNF25-mediated E-cadherin protein degradation

Zhao Huang[1#], Li Zhou[3#], Jiufei Duan[1#], Siyuan Qin[1], Jingwen Jiang[4], Haining Chen[5], Kui Wang[6], Rui Liu[7], Minlan Yuan[8], Xiangdong Tang[9], Edouard C. Nice[10], Yuquan Wei[1], Wei Zhang[8, 11*], Canhua Huang[1, 2*]

[1] Department of Biotherapy, Cancer Center and State Key Laboratory of Biotherapy, West China Hospital, Sichuan University, Chengdu, 610041, China.

[2] Frontiers Medical Center, Tianfu Jincheng Laboratory, Chengdu, 610212, China

[3] Key Laboratory of Molecular Biology for Infectious Diseases (Ministry of Education), Institute for Viral Hepatitis, Department of Infectious Diseases, the Second Affiliated Hospital, Chongqing Medical University, Chongqing, 400016, China.

[4] West China School of Public Health and West China Fourth Hospital, Sichuan University, Chengdu, 610041, China.

[5] Colorectal Cancer Center, Department of General Surgery, West China Hospital, Sichuan University, Chengdu, 610041, China.

[6] West China School of Basic Medical Sciences & Forensic Medicine, and State Key Laboratory of Biotherapy, West China Hospital, Sichuan University, Chengdu, 610041, China.

[7] State Key Laboratory of Oral Diseases, National Clinical Research Center for Oral Diseases, Research Unit of Oral Carcinogenesis and Management, Chinese Academy of Medical Sciences, West China Hospital of Stomatology, Sichuan University, Chengdu, 610041, China.

[8] Mental Health Center and Psychiatric Laboratory, the State Key Laboratory of Biotherapy, West China Biomedical Big Data Center, West China Hospital of Sichuan University, Chengdu 610041, China.

[9] Sleep Medicine Center, Department of Respiratory and Critical Care Medicine, Mental Health Center, Translational Neuroscience Center, and State Key Laboratory of Biotherapy, West China Hospital, Sichuan University, Chengdu, China.

[10] Department of Biochemistry and Molecular Biology, Monash University, Clayton, VIC, Australia.

[11] Medical Big Data Center, Sichuan University, Chengdu 610041, China.

[#] These authors contribute equally to this work.

* Correspondence: hcanhua@scu.edu.cn (C. Huang), weizhang27@scu.edu.cn (W. Zhang)

Supporting Information

Supporting figures

Figure S1

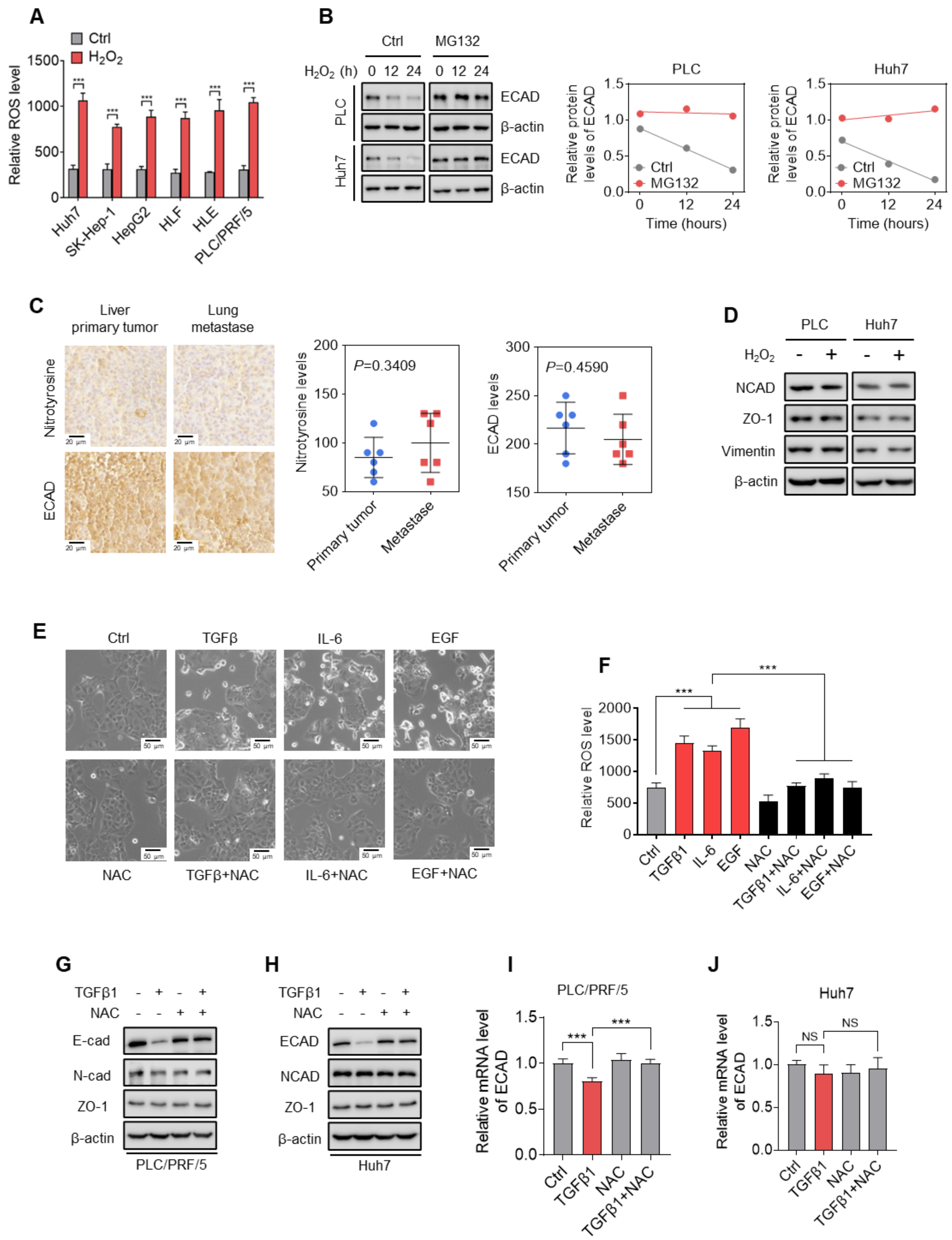

Figure S1. Oxidative stress causes E-cadherin protein repression in HCC. (A) ROS levels in Figure 1G were measured by fluorescence using the DCFH-DA method. (B) Western blot showing the protein expression of ECAD in HCC cells treated with 100 μM H_2O_2 for indicated time in the absence or presence of 25 μM MG132. (C) Immunohistochemistry assay showing the level of nitrotyrosine and ECAD in the primary tumor and metastatic tumor in Figure 1B. Scale bar, 20 μm. (D) Immunoblot assay showing the expression of indicated proteins in PLC and Huh7 cells with or without 100 μM H_2O_2 treatment for 24 hours. (E) PLC/PRF/5 cells were treated with 10 ng/ml TGFβ1, IL-6, EGF or 0.5 mM NAC for 72 hours, and the cell morphology was captured by microscopy. Scale bars, 50 μm. (F) ROS level in (E) was measured from fluorescence by the DCFH-DA method. (G-J) Western blot and qPCR assays showing the protein and mRNA levels of indicated genes in cells treated as (E). Data are mean ± SD from at least 3 independent repeats. *P*-value was determined by unpaired t test (A, C, F, I, J). *$P < 0.05$, **$P < 0.01$; ***$P < 0.001$. NS, no statistical significance.

Figure S2

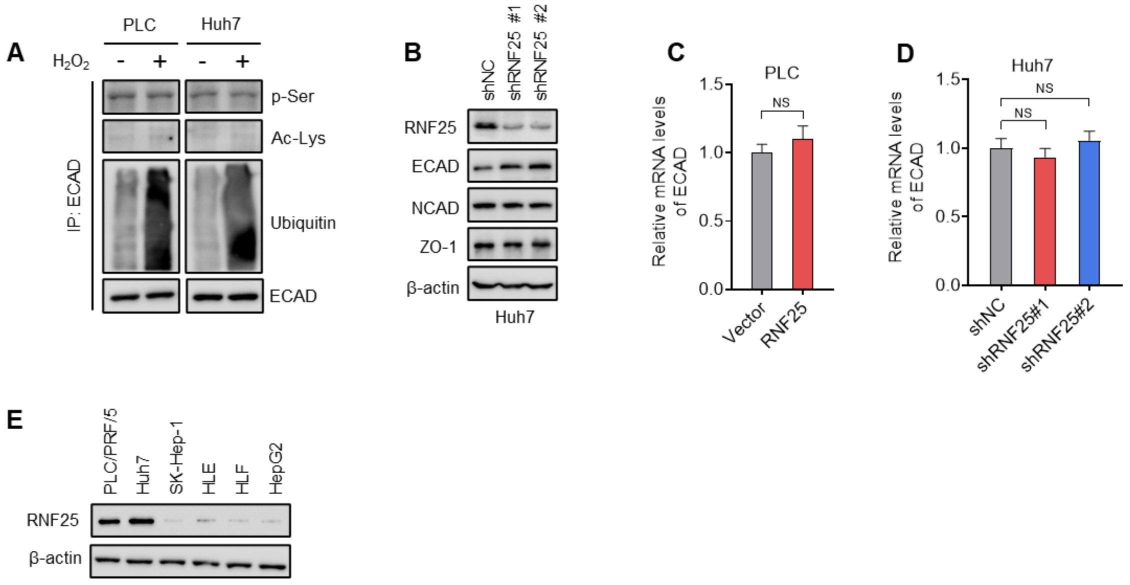

Figure S2. RNF25 is an E3 ligase of E-cadherin protein. (A) Immunoprecipitation assay showing the phosphorylation, acetylation and ubiquitination levels of ECAD protein in PLC and Huh7 cells

with or without 100 μM H_2O_2 treatment for 24 hours. p-Ser, phosphorylated serine. Ac-Lys, acetylated lysine. (B) Western blot showing the expression of indicated proteins in RNF25 knockdown cells. (C-D) The mRNA level of ECAD in indicated cells was determined by RT-qPCR. (E) Immunoblotting assay showing the protein level of ECAD in a panel of HCC cell lines. Data are mean ± SD from at least 3 independent repeats. *P*-value was determined by unpaired t test. NS, not significant.

Figure S3

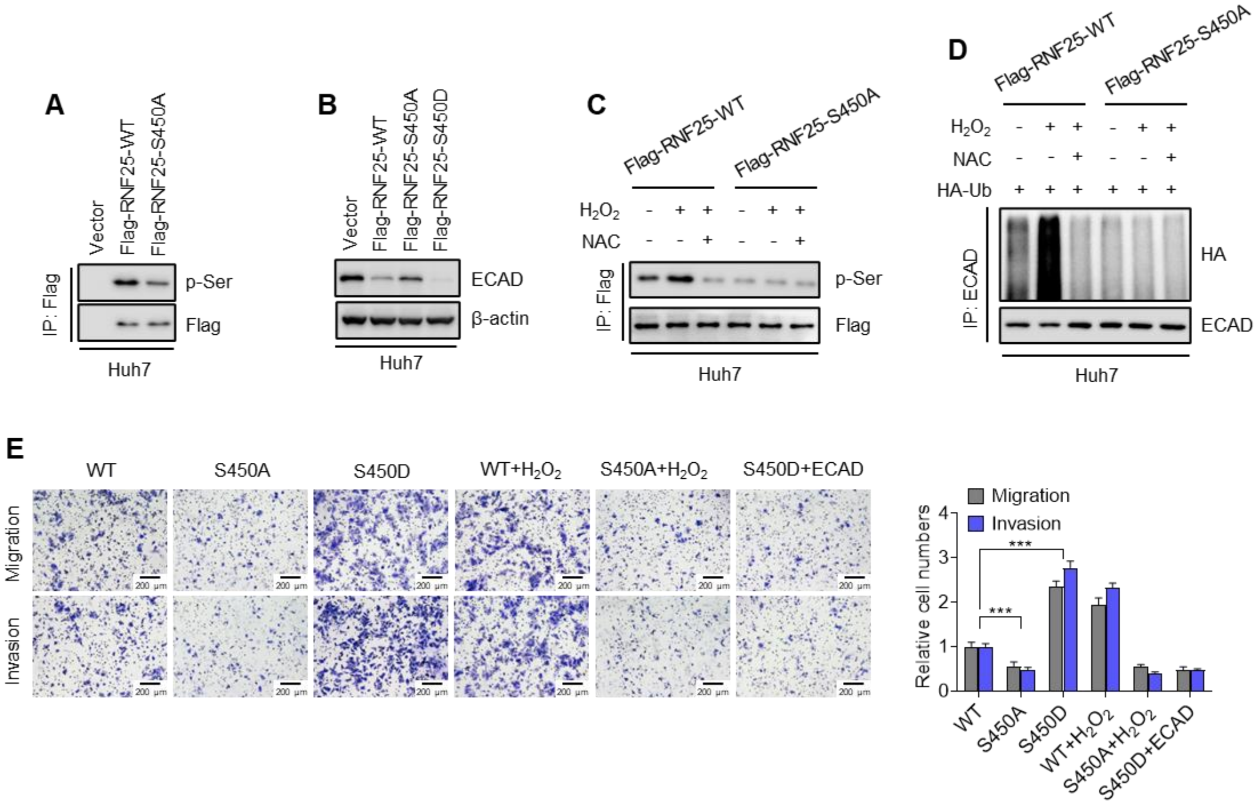

Figure S3. Phosphorylated RNF25 at Ser450 initiates E-cadherin protein degradation. (A) Immunoprecipitation assays showing the phosphorylation of wild-type and S450A mutated RNF25 in Huh7 stable cells. (B) Western blot showing the ECAD protein levels in Huh7 stable cells. (C) Immunoprecipitation assays showing the phosphorylation of indicated cells with or without the treatment of 100 μM H_2O_2 or 1 mM NAC for 1 hour. (D) Immunoprecipitation assays showing the ubiquitination of ECAD in indicated cells with or without the treatment of 100 μM H_2O_2 or 1 mM NAC for 24 hours. (E) Transwell assay showing the migration and invasion of Huh7-RNF25-WT, Huh7-RNF25-S450A and Huh7-RNF25-S450D cells with or without the overexpression of ECAD or the treatment of 100 μM H_2O_2. Scale bars, 200 μm. Data are mean ± SD from at least 3 independent repeats. *P*-value was determined by unpaired t test (E). **P* < 0.05, ***P* < 0.01; ****P* < 0.001.

Figure S4

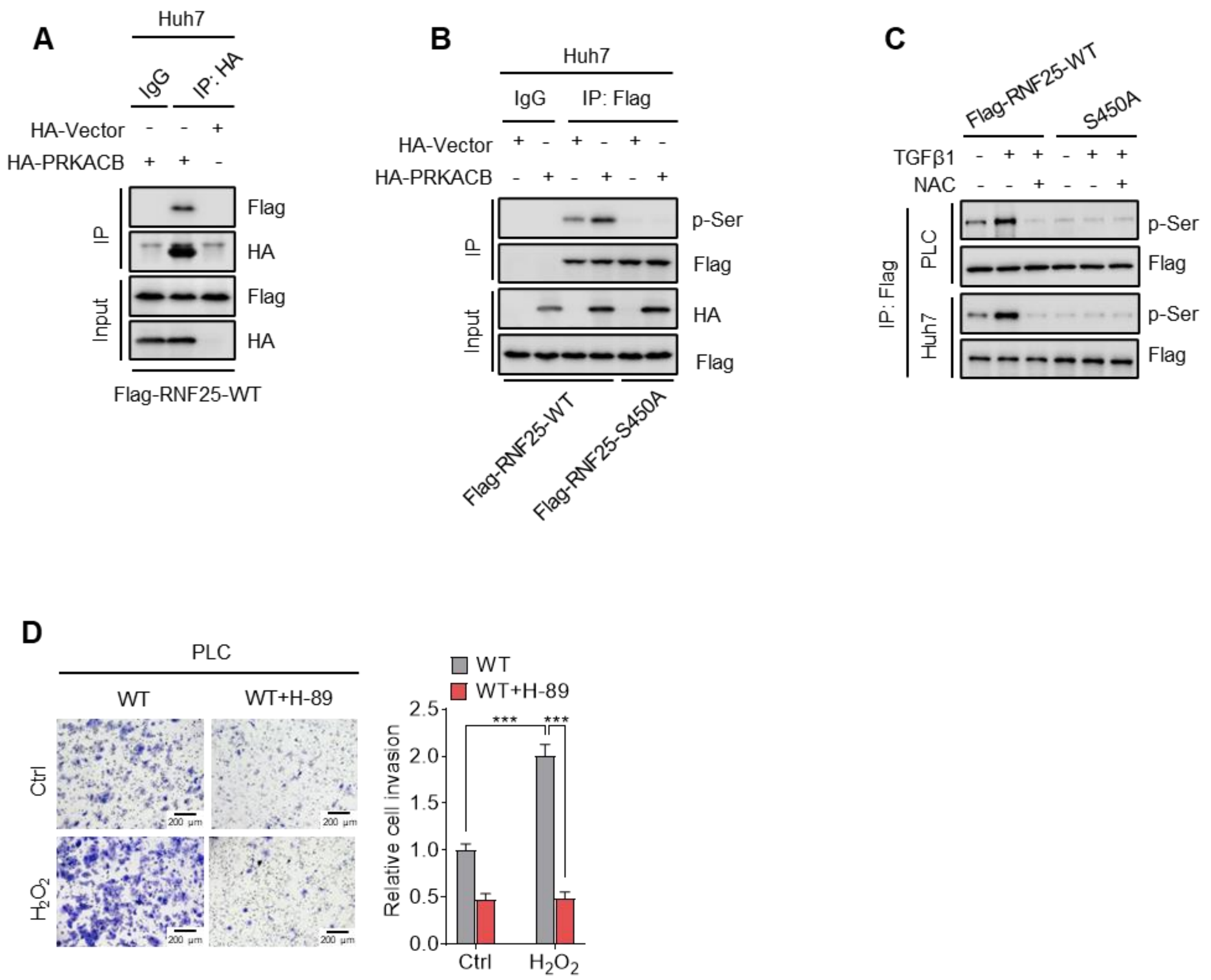

Figure S4. Protein Kinase A phosphorylates RNF25 at S450. (A) HA-tagged PRKACB was transiently expressed in Flag-RNF25-WT Huh7 cells, followed by detection of the interaction between PRKACB and RNF25 by co-IP assays. (B) Immunoprecipitation assays showing the phosphorylation of wild-type or S450A mutated RNF25 in Huh7 cells with or without transient overexpression of PRKACB. (C) Phosphorylation of wild-type or S450A mutated RNF25 was determined with or without the treatment of 10 ng/ml TGFβ1 or 0.5 mM NAC for 3 days. (D) Transwell assay showing the invasion of PLC-RNF25 cells treated with or without 100 μM H_2O_2 or 1 μM H-89. Scale bars, 200 μm. Data are mean ± SD from at least 3 independent repeats. *P*-value was determined by unpaired t test (D). **P* < 0.05, ***P* < 0.01; ****P* < 0.001.

Figure S5

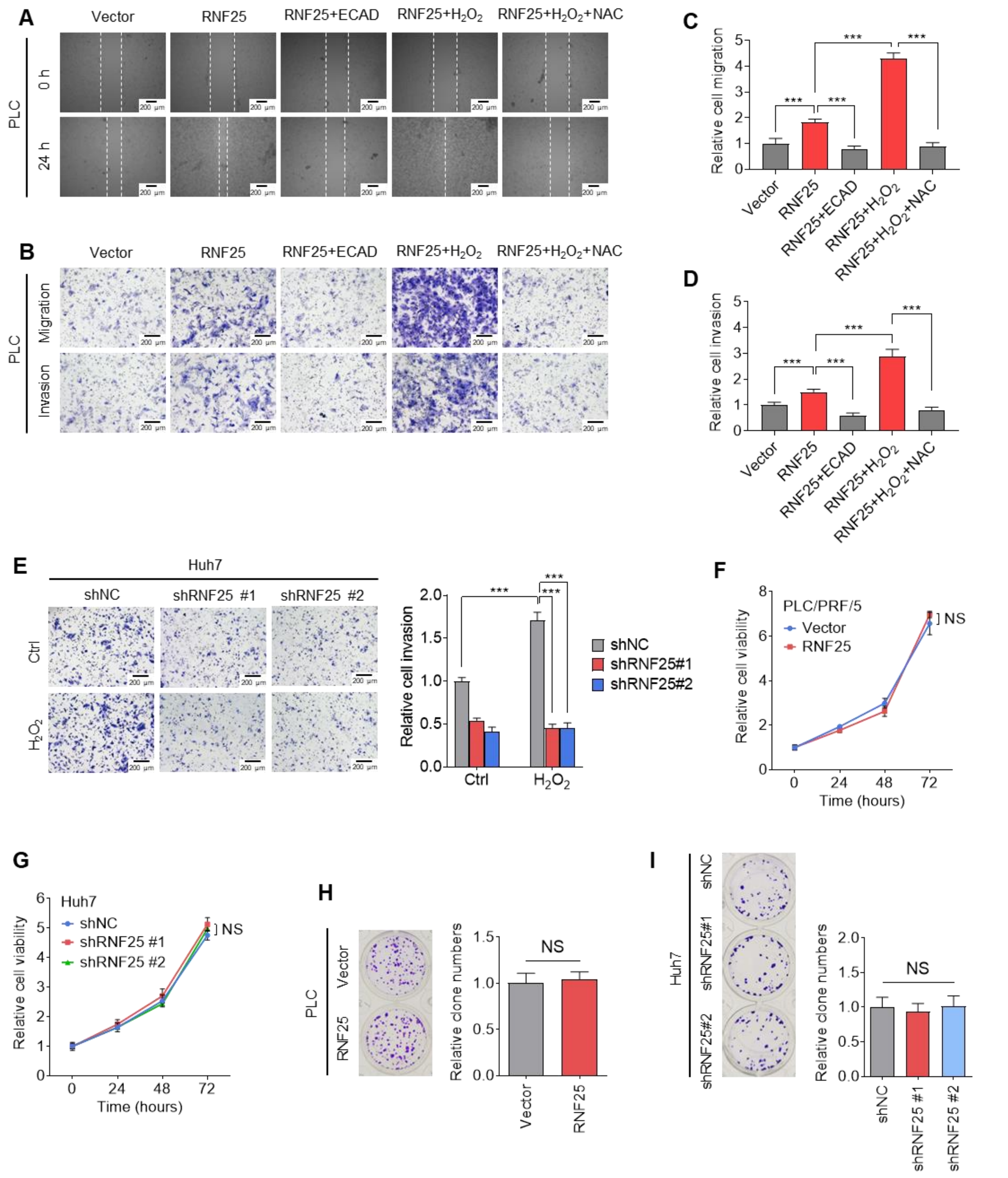

Figure S5. ROS-induced RNF25 phosphorylation degrades ECAD protein to promote HCC metastasis. (A) Wound healing assay showing the migration of PLC-Vector and PLC-RNF25 cells with or without the overexpression of ECAD or treatment with 100 μM H_2O_2 or 0.5 mM NAC. Scale

bars, 200 μm. (B-D) Transwell assay showing the migration and invasion of PLC-Vector and PLC-RNF25 cells treated as (A). Scale bars, 200 μm. (E) Transwell assay showing the invasion of Huh7 cells with or without RNF25 knockdown or H_2O_2 treatment. Scale bar, 200 μm. (F-I) MTT and colony formation assays showing the proliferation of HCC cells with or without the overexpression or knockdown of RNF25. Data are mean ± SD from at least 3 independent repeats. *P*-value was determined by unpaired t test (C, D, E, H, I) and Two-way ANOVA (F, G). *$P < 0.05$, **$P < 0.01$; ***$P < 0.001$.

Figure S6

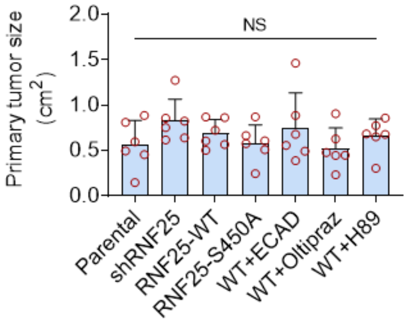

Figure S6. ROS-PKA-RNF25-ECAD axis does not obviously change primary tumor growth in HCC. Quantification of primary tumor size in Figure 6A. NS, not significant. Data are mean ± SD from at least 3 independent repeats. *P*-value was determined by unpaired t test.

Figure S7

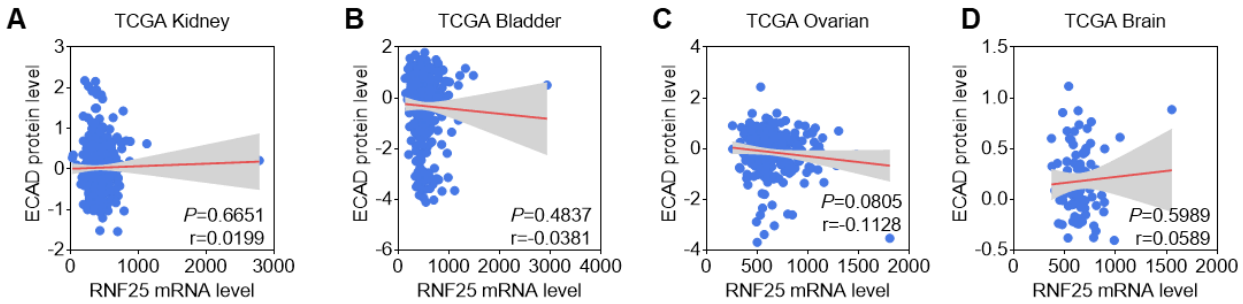

Figure S7. RNF25 is overexpressed in human HCC tissue and associated with poor prognosis. (A-D) The correlation between RNF25 mRNA level and ECAD protein level in different cancer types was evaluated using the TCGA dataset. *P*-value was determined by Pearson correlation test.

Supporting tables

Table S1. Top 10 interactors of ECAD were ranked by mass spectrometry (MS) intensity.

Rank	Gene	UniProt ID	Protein
1	HNRNPK	P61978	Heterogeneous nuclear ribonucleoprotein K
2	RNF25	Q96BH1	E3 ubiquitin-protein ligase RNF25
3	PDIA3	P30101	Protein disulfide-isomerase A3
4	HSPD1	P10809	60 kDa heat shock protein, mitochondrial
5	KRT1	P04264	Keratin, type II cytoskeletal 1
6	KRT9	P35527	Keratin, type I cytoskeletal 9
7	KRT10	P13645	Keratin, type I cytoskeletal 10
8	ERO1A	Q96HE7	ERO1-like protein alpha
9	CCT6A	P40227	T-complex protein 1 subunit zeta
10	MTHFD1	P11586	C-1-tetrahydrofolate synthase, cytoplasmic

Table S2. Top 10 interactors of RNF25 were ranked by mass spectrometry (MS) intensity.

Rank	Gene	UniProt ID	Protein
1	PRR32	B1ATL7	Proline-rich protein 32
2	hCG_1982563	A0A024R5Q9	HCG1982563
3	KRT1	P04264	Keratin, type II cytoskeletal 1
4	PRSS1	P07477	Serine protease 1
5	EEF1A1	P68104	Elongation factor 1-alpha 1
6	PRKACB	P22694	cAMP-dependent protein kinase catalytic subunit beta
7	CCDC85A	Q96PX6	Coiled-coil domain-containing protein 85A
8	LDHA	P00338	L-lactate dehydrogenase A chain
9	SOD1	P00441	Superoxide dismutase [Cu-Zn]
10	RL31	P62899	Large ribosomal subunit protein eL31
